# Human *Oestrus* sp. Infection, Canary Islands

**DOI:** 10.3201/eid1306.060882

**Published:** 2007-06

**Authors:** Marion Hemmersbach-Miller, Rita Sánchez-Andrade, Alicia Domínguez-Coello, Adnan Hawari Meilud, Adolfo Paz-Silva, Cristina Carranza, Jose-Luis Pérez-Arellano

**Affiliations:** * Hospital Ntra. Sra. de los Reyes, Valverde. El Hierro, Spain; † Hospital San Roque Maspalomas, Gran Canaria, Spain; ‡ Santiago de Compostela University, Lugo, Spain; § Veterinarian of the Cooperativa de Ganaderos de El Hierro, El Hierro, Spain; ¶ University of Las Palmas de Gran Canaria, Las Palmas de Gran Canaria, Spain

**Keywords:** Oestrus, eosinophilia, serologic diagnosis, letter

**To the Editor:** Myiasis due to *Oestrus ovis* is a well known zoonosis that affects a variety of animals. Human myiasis has also been described and affects mainly persons in rural areas such as shepherds (*1*) and farmers (*2*). Although this disease has been reported in both humans and mammals in Spain (*3*,*4*), no human case has been described on the Canary Islands. We describe what we believe is the first confirmed case on the islands and discuss the potential utility of serologic diagnosis for this disease.

A 55-year-old farmer from the island of El Hierro, with a medical history of hypercholesterolemia, Q fever, and murine typhus, but currently not being treated, consulted a physician in August 2005 concerning a wormlike sensation in his nose and sinuses that had lasted 2 days. Three days before noticing this sensation, he had been working in his neighbor’s barn, when he noticed that a passing fly “dropped” something in his nose. He also reported sneezing and watery rhinorrhea. These symptoms were self-treated with nasal anticongestants, which provided temporary relief. He finally sought medical attention when a severe cough developed and the wormlike sensation extended to his throat.

On physical examination, the patient’s vital signs were normal, although a turbinate hypertrophy and mild redness of the throat were noted. No foreign objects or insects were seen on otorhinolaryngologic examination. The patient’s blood count showed 8,480 leukocytes/μL with 6.1% (520/μL) eosinophils. Because of his stated symptoms, myiasis was suspected, and symptomatic treatment was started, consisting of antihistamines, nasal anticongestants, cough suppressants, and asphyxiant methods, i.e., swallowed olive oil. The patient was monitored closely and had complete remission of his symptoms after 6 days. No relapse has occurred.

In the meantime, we discovered that a serologic test for *O. ovis* was available (*5*). We requested and obtained a convalescent-phase serum sample from the patient on day 14 of his illness. Blood was also obtained from different “healthy” animals in the patient’s neighborhood, including 2 dogs, 4 sheep, and 5 goats. This serologic assay had not previously been used in testing humans. Excretory and secretory antigens from *O. ovis* L2 (OL2ES) were obtained as previously described (*6*), and samples were analyzed by an immune enzymatic assay technique (*7*). Appropriate testing with different dilutions of the antigens, sera, and immunoconjugates was conducted. Immunoglobulin G (IgG) was detected in the patient, sheep, goats, and dogs following a similar protocol. OL2ES concentrations were 1, 1, 3, and 5 μg/mL, respectively. Serum samples were diluted 1:100 for the patient and the dogs and 1:50 for the goats; immunoconjugates were diluted 1:1,500 for all species. *O. ovis* IgG was found in the patient’s sera, as well as in sera of the 2 dogs, 2 of 4 sheep, and all 5 goats (Table).

**Table:** Results and interpretation, *Oestrus* sp. infection, Canary Islands*†

Human		Dogs		Sheep		Goats	
OD	Interpretation	OD	Interpretation	OD	Interpretation	OD	Interpretation
0.658	Positive	0.677	Positive	0.639	Positive	0.838	Positive
		0.824	Positive	0.685	Positive	0.535	Positive
				0.226	Negative	0.594	Positive
				0.187	Negative	0.673	Positive
						0.622	Positive

*Results are expressed as optical density (OD), and interpretation (positive/negative) was made by using the following cut-offs: in sheep: 0.369 (0.1718 + 3 × 0.066); goats: 0.406 (0.211 + 3 × 0.065); human 0.32 (0.17 + 3 × 0.049); dogs: 0.493 (0.37 + 3 × 0.041). †One sample of positive and negative control samples was added to each plate. Sheep and goat sera from animals with a known history of *O. ovis* exposure were used. When positive sera were not available (human and dogs), we used only negative sera, and the cut-off was estimated as the mean OD of the negative sera plus 3 SDs (*7*).

Human infection by *O. ovis* is generally considered to be an accidental occurrence (*8*). This case confirms, however, that myiasis caused by *O. ovis* must be considered in the differential diagnosis of a patient with typical symptoms and eosinophilia. Most farmers in this area have reported similar symptoms. Most, however, do not seek medical attention because they prefer to use homemade remedies, such as topical oil.

The diagnosis of oestrosis is usually made by direct visualization of the larvae, since the most frequent symptoms are pharyngeal myiasis and ophthalmomyiasis. Immunodiagnostic methods, however, could be a viable alternative to the clinical examination when no larvae are directly seen but a high degree of suspicion exists. The ELISA was noted to have a sensitivity of 96.1% and a specificity of 55.8% (positive predictive value of 86.7% and negative predictive value of 82.8%) in various investigations made with sheep and goats (*6*).

Although allergic symptoms are frequent in animals, the pathophysiologic process seems to be different in humans (*8*). Nevertheless, other authors have also described coughing and sneezing (*1*), probably attributable to irritation of the mucosa. In animals, a primary peak in eosinophil numbers has been noted 4 days after infection with a primary increase 48 hours after infection (*9*). In humans this pattern has not been described, but we did note a mild eosinophilia that disappeared after the patient recovered from his symptoms.

Outcome of the disease in humans is generally benign. Treatment includes removal of the larvae and, in some cases, prevention of local infections. Ivermectin has also been found useful in animal and human infections (*10*).

To our knowledge, this is the first case of human oestrosis on the Canary Islands, as well as the first human case described with eosinophilia. Physicians should be aware of the possibility of this disease in our region and of the fact that a serologic test is available for its diagnosis.
